# Should tuberosities be reattached in patients over 80 years old treated with reverse shoulder arthroplasty after proximal humeral fractures?

**DOI:** 10.1177/17585732261423483

**Published:** 2026-02-27

**Authors:** Pablo Luque-Amo, Alberto Izquierdo-Fernández, Jose Carlos Miñarro, Fernando Santana, Carlos Torrens

**Affiliations:** 1 Department of Orthopaedic Surgery, 16501 Reina Sofía Hospital, Cordoba, Spain; 2 Orthopaedics and Trauma Department, 16546 Parc de Salut Mar. Universitat Autónoma de Barcelona, Barcelona, Spain

**Keywords:** Reverse shoulder arthroplasty, proximal humerus fracture, tuberosities, outcomes, elderly, constant score

## Abstract

**Introduction:**

Reverse Shoulder Arthroplasty has shown better tuberosity healing rate when compared to hemiarthroplasty, but little information is known on how ageing affects tuberosity healing rate. The aim of this study was to evaluate the clinical and radiological outcomes of reverse shoulder arthroplasty after proximal humeral fracture in patients over 80 years old.

**Methods:**

We performed a retrospective study including 75 consecutive patients treated with a RSA because of a PHF. Patients were divided in two groups according to their age. Constant Score was recorded at 2 years follow-up. Healing rate of greater tuberosity was assessed on plain X-rays exams.

**Results:**

No differences were noted in tuberosity healing between groups. Functional outcomes of patients over 80, measured according to the Constant Murley Score, were slightly better when tuberosity healed without reaching significance. Gender, laterality, glenosphere size and ASA score had no effect in tuberosity healing.

**Conclusion:**

Tuberosity healing in RSA for PHF is not impaired in patients over 80 years old. Functional results in patients over 80 with healed tuberosities are slightly better than in those without healing.

## Introduction

Reverse shoulder arthroplasty (RSA) offers an established treatment option in rotator cuff arthropathy. Pain and clinical scores have effectively been improved in those patients in whom nonoperative treatment has failed.^1,2^ Treatment of rotator cuff arthroplasty was significantly improved with the design of the reverse shoulder implant, obtaining improved function, motion, and pain compared to previous treatments.^3^ Indications have recently spread to treat acute fractures, malunions, tumors or revision surgery.

Proximal humerus fractures (PHF) represent the second-most-common fracture in the upper limb, accounting for 5% of fractures in adults.^4^ The management of PHF in elderly patients is still controversial and there is no consensus on literature about the ideal treatment option. Classically, when the vascularization of the humeral head was compromised, hemiarthroplasty (HA) was the most common treatment option. However, clinical outcomes of hemiarthroplasties used in complex PHF are unpredictable,^5,6^ mainly because of the high rate of complications related to the tuberosities, as malposition or resorption.^6^

RSA was initially designed to treat cuff-deficient shoulders, as its biomechanical design relies on the deltoid muscle to generate motion. Therefore, when used in PHF, tuberosity healing and position might not impair clinical results as dramatically as when using a HA. There are studies which have recommended not reattaching the tuberosities when dealing with PHF,^7,8^ although other complications have been described, such as instability or implant loosening when tuberosities are absent or not healed.^9^

Recent data suggests improved clinical outcomes when tuberosities heal properly, with greater external rotation restoration.^10,11^ However, healing rates vary greatly in the literature^12,13^ and it seems to be impaired in elderly patients as age increases. There is a paucity of information on how aging affects tuberosity healing.^14^

Our aim is to determine if there are differences in the healing rate of tuberosities reattached in patients over 80 years when compared with patients under 80, when treated with an RSA after PHF. Also, we want to assess if tuberosity healing results in improved functional outcomes.

## Material and methods

A retrospective comparative cohort study was designed from our prospective database. The inclusion criteria were: (a) patients over 65 years old with (b) a displaced 3- or 4-part PHF as classified by Neer with a (c) minimum follow-up of 2 years after RSA. The exclusion criteria were: (a) fractures presented to clinic after 30 days, (b) previous injuries of the same upper limb or (c) lost to follow-up.

From January 2014 to December 2019, 82 consecutive patients treated with an RSA because of a PHF were included. Four were lost to follow-up before the 24 months’ deadline and three died during the first year without direct relation to the ongoing surgical procedure. This left a final cohort of 75 patients (75 shoulders) (Figures 2 and 3).

**Figure 1.**
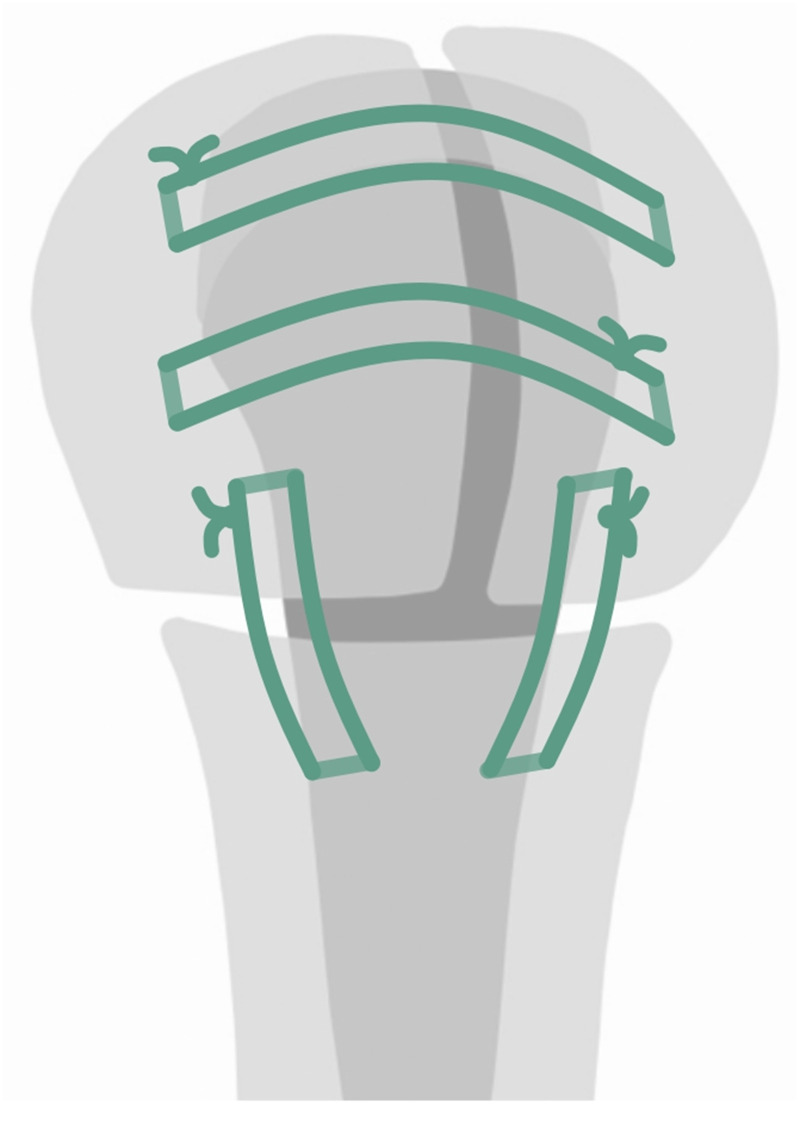
Tuberosity fixation technique.

All surgeries were performed by one senior surgeon, specializing in shoulder surgery, with the same implant (Delta Xtend; DePuy-Synthes, Warsaw, IN, USA), using a standard technique in the beach-chair position. The anterosuperior approach was used in all cases and, after fracture exposition, tuberosities were mobilized and grasped with a heavy nonabsorbable braided suture (No. 5 Ethibond; Ethicon, Somerville, NJ, USA). The head was therefore removed and the glenoid exposed and prepared, conserving as much subchondral bone as feasible. The humeral component was always cemented and placed in a neutral position (0° degrees of retroversion). In all cases, tuberosities were reattached as anatomically as possible with the following configuration: two horizontal and two vertical sutures (from diaphysis to greater tuberosity and from diaphysis to lesser tuberosity) with previously used Ethibond. The supraspinatus tendon was excised in all cases (Figure 1).

**Figure 2.**
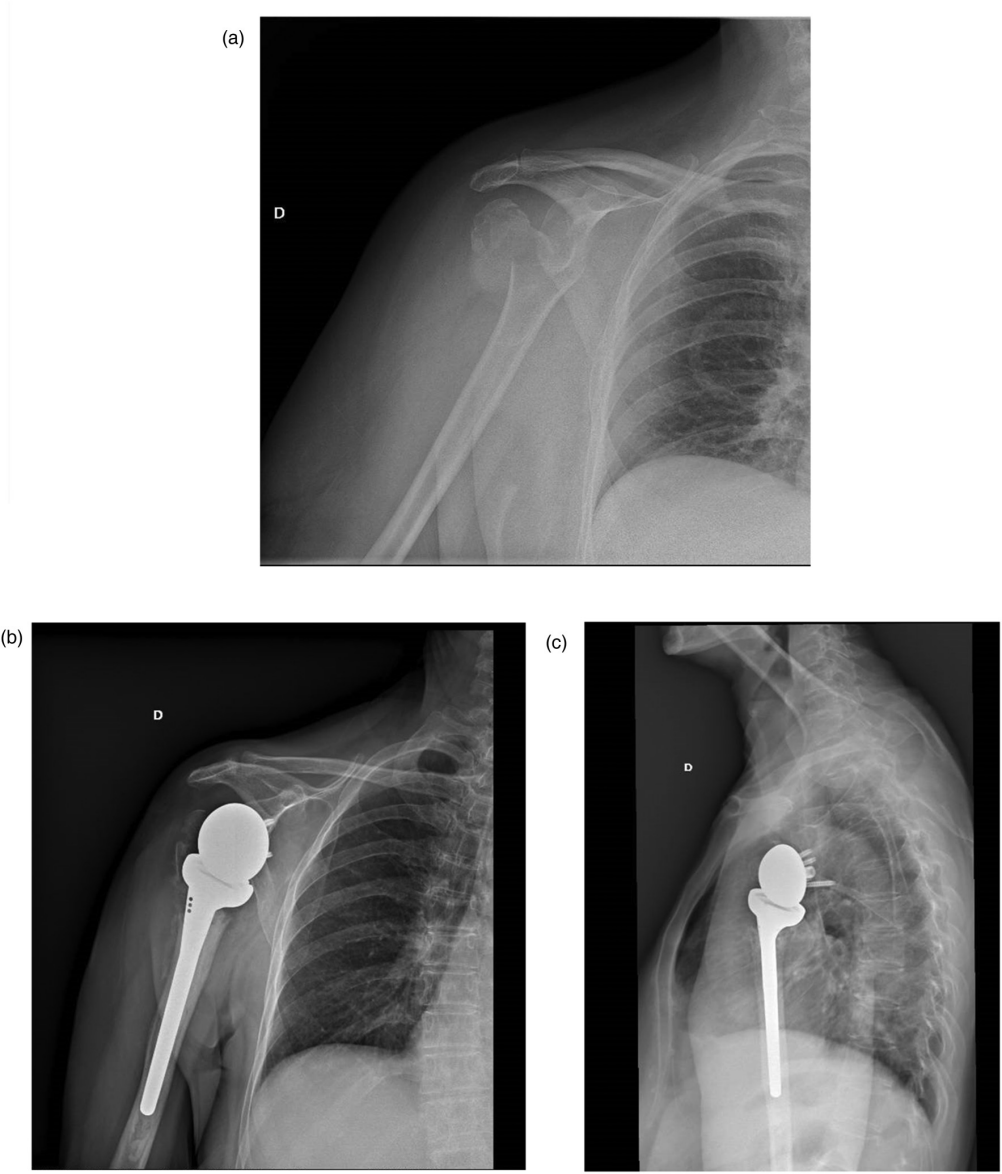
81-year-old female who suffered a 4-part proximal humerus fracture, anteroposterior radiograph same day of injury (A), anteroposterior (B), and transthoracic (C) views during follow-up.

**Figure 3.**
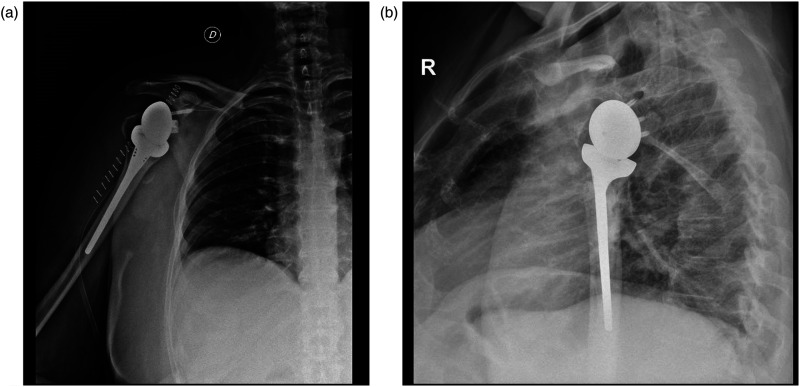
75-year-old male who underwent reverse shoulder arthroplasty after a complex proximal humerus fracture: anteroposterior (A) and transthoracic (B) radiographs immediately after surgery.

Postoperatively, a suction drain was left during 48 h after which the patient was released to outpatient follow-up. The arm was placed in a neutral rotation sling for 3 weeks, and pendulum exercises were explained and initiated after 3 weeks followed by passive elevation and rotations as progression occurred. Return to normal previous activities was allowed 3 to 6 months postoperatively.

The primary outcome measure on this study was to compare functional outcomes and tuberosity healing rate between patients over and under 80 years old. Shoulder function was finally assessed at 24 months using the absolute Constant-Murley Score (CMS); routine radiographic analysis included anteroposterior and lateral views and were obtained at 1,3,6,12 and 24 months after surgery. Healing rate of greater tuberosity was evaluated by an independent external radiologist on the last plain X-Ray exam, as described by Boileau.^6^ GT was considered to be anatomically healed when it was visible on the lateral side on the stem, showing continuity with the diaphysis and not lower than 5 mm from the prosthetic head. Influence of both age and gender in the healing of tuberosities was also analyzed. Secondary variables such as type of fracture, time until surgery, glenosphere and polyethylene size were recorded and analyzed.

### Statistical analysis

Sample size was calculated accepting an alpha risk of 0.05 and a beta risk of less than 0.2 in a bilateral contrast, it takes 36 subjects in the group 1 (under 80) and 18 in the group 2 (over 80) to detect a statistically significant difference between two proportions, that for group 1 is expected to be 0.65 and for group 2 to be 0.95. A follow-up loss rate of 20% has been estimated. The ARCSINUS approach has been used.

Statistical analysis was performed with SPSS version 22 (IBM, Armonk, NY, USA). Descriptive statistical figures for continuous variables were presented using mean values and standard deviation. Categorical data were presented as frequencies and percentages. Normal distribution of variables was assessed using the Shapiro-Wilk test. To compare continuous parametric data in groups we used the Student t test, the c2 test for categorical parametric data and the Mann‒Whitney for nonparametric continuous data. The significance level was set at 0.05.

## Results

Fifty patients under 80 years and 25 years older than 80 years underwent RSA to treat acute displaced PHF. There were 62 females (82.7%) and 13 males (17.3%), with a mean age of 76.38 (range: 65–90 years). The delay to surgery from the day of injury averaged 11.37 days (range: 0–29 days).

There were no significant differences between groups according to gender, laterality, delay to surgery, fracture classification, glenosphere size or polyethylene size. Epidemiologic data are summarized in Table 1.

**Table 1.** General epidemiological characteristics according to age.

Variable			
	Under 80 (*n* = 50)	80 or above (*n* = 25)	*p* value
Gender, male/female	11/39	2/23	.131
Injured side, left/right	31/19	12/13	.247
Neer classification, 3- and 4-part	19/31	5/20	.268
Average time to surgery (days)	11.45	11.22	.904
Glenosphere size, 38/38 ECC/42	7/10/33	4/6/15	.916
Polyethylene size, 3/6/9 mm	25/21/4	7/14/4	.236

### Greater tuberosity healing rate

Overall, greater tuberosity consolidation was achieved in 57 patients (76%). In patients under 80 years, the consolidation rate was 78% (39 patients) while in patients over eighty it was 72% (18 patients). This difference was not statistically significant (*p =* .069). Gender did not influence the consolidation of the greater tuberosity (*p =* .865). Glenosphere size (*p =* .796) and polyethylene used (*p =* .497), did not significantly influence greater tuberosity healing.

### Functional results

Patients under eighty had significant better outcomes according to total CMS, strength, forward elevation and abduction when compared with patients over eighty. Postoperative functional outcomes at two-year follow-up are summarized in Table 2.

**Table 2.** Postoperative functional and radiographical outcomes according to age.

Variable			
	Under 80	80 or above	*p* value
Mean Constant-Murley score	63.55	57.23	.017
Postoperative pain	12.3	13.3	.345
Strength	7.77	5.55	.001
Daily life activities	17.27	16.2	.161
Forward elevation	7.33	5.87	.022
Abduction	7	5.73	.022
External rotation at 0°	5.67	5.07	.544
Internal rotation	5.87	5.07	.772
Greater tuberosity healing (%)	39 (78%)	18 (72%)	.07

Significant but not clinically relevant differences were noted in mean final Constant Score in patients under the age of 80 (63.55) when compared to patients over 80 (57.23) (*p =* .017).

Patients with tuberosity healing had better outcomes when compared with those not healed, but significance was not reached. The difference in functional results between patients depending on tuberosity healing is shown in Table 3.

**Table 3.** Postoperative functional outcomes according to tuberosity healing.

Variable			
	Consolidated GT	Non-consolidated GT	*p* value
Mean Constant-Murley score	61.53	61.21	.650
Mean postoperative pain	12.42	13.22	.705
Mean strength	7.46	6.52	.263
Mean “activities daily living” (ADL)	16.94	16.78	.854
Mean forward elevation	6.95	6.83	.277
Mean abduction	6.61	6.44	.885
Mean external rotation at 0°	5.36	4.34	.209
Mean internal rotation	5.51	4.05	.173

Functional outcomes of patients over 80 were slightly better when tuberosities healed, although statistical significance was not reached, as shown in Table 4.

**Table 4.** Postoperative functional outcomes in the “80 years or above” group.

Variable			
	Consolidated GT	Non-consolidated GT	*p* value
Mean Constant-Murley score	58.07	51.5	.471
Mean forward elevation	6.77	5.1	.486
Mean abduction	5.84	5	.665
Mean external rotation at 0°	5.07	4.8	.646
Mean internal rotation	5.23	4.1	.516

### Complications

There was only one dislocation in a 73-year-old male after an accidental fall three months after surgery. Satisfactory closed reduction was performed in the emergency department followed by discontinuous sling immobilization for 3 weeks. Tuberosities healed properly in this patient and no further episodes of dislocation were recorded to date. No other complication was recorded.

## Discussion

Reverse shoulder arthroplasty (RSA) has shown improved functional outcomes and greater tuberosity consolidation in patients suffering a PHF, compared to HA.

Several studies have addressed the need to reattach the tuberosity and its repercussion on functional outcomes. Cuff et al. obtained an 83% tuberosity healing rate and they showed non-statistically significant differences between groups, except for external rotation which was improved in the consolidated group.^13^ A different study from Sebastiá-Forcada et al.,^15^ which compared HA to RSA in treating PHF, concluded that tuberosity healing did not affect clinical outcomes after analyzing the group of fractures treated with the reverse arthroplasty. More recently, Ohl et al.^16^ compared three cohorts of RSA in PHF depending on surgical tuberosity management (excision vs conservation) and they showed better clinical results when tuberosities healed anatomically. Mixed results have been reported in other clinical studies.^10,12,17^

In studies with longer follow-up periods, the tuberosities healing rate appears to decrease. Allio et al.^18^ reported a 37% resorption rate with more than five years after surgery and Izquierdo-Fernández et al.^19^ reported a 31.25% non-healing rate with a follow up of at least 7 years. However, this decrease does not appear to correlate with poorer clinical outcomes, as reported in these clinical series.

We need well-designed prospective, randomized clinical trials to clarify the relation between tuberosity fixation and clinical outcomes. Although that evidence is still missing, our study shows that age should not influence this decision, since consolidation rate is the same in.

Our study was undertaken to investigate the correlation of age and tuberosity healing, specifically considering 80 years as the cut-off point being the most extreme value likely to influence clinical results. We believe that the definition of an elderly population has been vaguely described in the literature and this leads to heterogeneous results based on the definition, as described by Lopiz et al.^14^ The most relevant finding of our study is that we confirmed our main hypothesis: tuberosity healing is not impaired in patients over 80 years old; thus age should not be considered as a decision-making variable. This is, to our knowledge, the first study to address directly relationship between specific age and tuberosity healing.

The consolidation rate shown in our population (76%) can be considered similar to what has previously been published^20–23^; and this rate is well above what has been described with HA.^24^ Recent trends to enhance tuberosity healing may increase consolidation rates, including bone grafting and specific humeral stems, although none of these were used in our study, except for excision of the supraspinatus, which has been shown to decrease the tension between the sutures.^25^

In terms of functional outcomes, several studies have proven that RSA is superior to HA after complex PHF. Similar results arose after comparing RSA with conservative treatment in older adults in a recent study by Chivot et al.,^26^ although clinical difference for the CMS was narrowly achieved.^27,28^ Our data suggest that clinical outcomes are slightly better in the younger population; nevertheless, clinical significance was hardly reached as the difference accounted only for 6.2 points in the CMS. In contrast, Rivera et al.^29^ found a 15-point CMS differential between patients older and younger than 75 years of age. It should be noted, however, that the overall CMS values in their serie is substantially higher than those typically reported in the literature. Therefore, we believe that patients above eighty, as long as they are active and fit to undergo surgery, can achieve good results after RSA for PHF.

Some studies showed improved shoulder rotation in patients with healed tuberosities after RSA, mostly external rotations.^21^ In a recent systematic review,^22^ from six studies (reporting about forward flexion and external rotation) five of them showed significant improvement in external rotation and three of them in forward flexion. Our results showed a subtle, not statistically relevant improvement in forward flexion, abduction and external rotation in patients with healed tuberosities. However, when addressing these differences related to functional outcomes, no significant improvement in ROM or CMS was found.

### Limitations and strengths

There are some limitations on this study. First, we conducted a single-institution retrospective study with no control group although data were recorded prospectively. Secondly, we assessed tuberosity healing in standard radiographs in two planes evaluated by an external radiologist. Finally, no healthy group was evaluated, nor any age-adjusted score was used to increase the external validity of our study.

Our study also has several potential strengths. We collected a well-defined cohort (82 patients older than 65 years with a 3- or 4-part PHF), and performed a follow-up of 24 months, both clinically and radiographically. Moreover, we used the same surgical procedure in all cases (same surgeon, same instrumentation, same implant and same technique) and ensured the same postoperative protocol for all patients.

## Conclusions

Our results confirm the main hypothesis that tuberosities healing in elderly patients are not impaired after RSA in PHFs; therefore, age should not be considered a contraindication for tuberosities fixation. However, we were unable to confirm that tuberosity consolidation improved functional outcomes after RSA. Further prospective studies are needed to clarify if it is worth reattaching the tuberosities in RSA after a PHF.
